# Postoperative permanent section evaluation of Mohs micrographic surgery debulk specimens does not result in upstaging of cutaneous squamous cell carcinoma compared to stage at the completion of surgery: A retrospective case series

**DOI:** 10.1016/j.jdin.2024.01.005

**Published:** 2024-01-20

**Authors:** Andrea Gilmore, Brianna Castillo, Nicholas Golda, Kara Braudis, Emily Smith

**Affiliations:** a Department of Dermatology, University of Missouri Columbia, Columbia, Missouri; b Department of Dermatology, University of Arizona, Tuscon, Arizona; c US Dermatology Partners, Lee’s Summit, Missouri; d Department of Dermatology, Saint Louis University, St. Louis, Missouri

**Keywords:** dermatopathology, (high-risk) squamous cell carcinoma, Mohs micrographic surgery

## Abstract

**Background:**

Mohs micrographic surgery (MMS) is used for the treatment of high-risk cutaneous squamous cell carcinoma (cSCC). MMS examines the surgical margins in real time and does not commonly examine the central component of the tumor.

**Objective:**

To determine if debulk specimens provide additional details relevant to tumor staging not gained from routine MMS.

**Methods:**

A retrospective chart review of debulk specimens taken during MMS for cSCC was performed. Dermatopathology reports were analyzed and tumors were staged using Brigham and Women’s Hospital and American Joint Committee on Cancer’s 8th edition staging systems.

**Results:**

Permanent section evaluation of debulk specimens did not result in clinically meaningful information for staging that could not be gained from MMS layers or initial biopsy analysis.

**Limitations:**

A single institution, and a small sample size of 39 tumors.

**Conclusions:**

Evaluation of debulk specimens during MMS may not always be an effective use of time or health care resources.

**Capsule Summary**

• Little is known about the staging effect of the evaluation of debulk specimens obtained during Mohs micrographic surgery for cutaneous squamous cell carcinoma.
• Permanent section evaluation of debulk specimens did not result in clinically meaningful changes in the cutaneous squamous cell carcinoma staging and may only have use in limited settings.

## Introduction

Mohs micrographic surgery (MMS) is the preferred treatment option for cutaneous squamous cell carcinoma (cSCC) at a high risk of recurrence or metastasis. MMS visualizes 100% of margins, and therefore results in a cure rate as high as 97%.^1^, ^2^, ^3^ However, because the emphasis is on the surgical margins, the central portion of the removed tumor may be curetted or discarded and not examined by the Mohs surgeon or a pathologist. Debulking is often performed to aid in defining subclinical margins and flattening the first Mohs layer for better visualization.^4^ This procedure, however, may result in a missed opportunity to identify histopathologic parameters that may modify tumor staging and patient management. There is limited information on the usefulness of evaluation of tumor debulk specimens.^5^, ^6^, ^7^, ^8^, ^9^

Mcllwee et al^5^ examined the use of debulk specimens during MMS for cSCC and found that intraoperative analysis of frozen specimens resulted in upstaging and identification of high-risk features that were not seen on biopsy or Mohs stages. Kyllo et al^6^ also examined frozen sections of both the debulk specimen and first Mohs stage of nonmelanoma skin cancer and found a significant portion were upgraded to more aggressive subtypes. Although frozen specimens do provide a quick and effective way for the tissue to be examined under the microscope, permanent specimens are considered the gold standard.^10^ Concordance rates between frozen and permanent sections have been reported to range from 70%-90%.^10^ This 10%-30% rate of discrepancy between frozen and permanent sections could lead to significant differences in tumor staging and subsequent management for patients. Nemeh et al^9^ examined permanent tissue sections from cSCC debulk specimens during MMS and found that tumors were upstaged 18% of the time on debulk analysis. In this study we aim to determine further information about the rate of upstaging of cSCC during MMS when debulk specimens are sent for permanent tissue section evaluation by a dermatopathologist.

## Methods

A retrospective chart review was performed at The University of Missouri Columbia Dermatologic Surgery Unit (Columbia, MO). The study was approved by the institutional review board at The University of Missouri—Columbia. Subjects included all adult patients who had undergone outpatient MMS for the treatment of cSCC with debulk specimens sent for permanent sections. The dermatopathology laboratory interface system and MMS biopsy log were queried for the word debulk from the dates of January 1, 2019, to June 30, 2021. For those that underwent MMS with debulk specimens of cSCC in the database, the patients’ electronic medical records were reviewed for cSCC debulk histopathologic and clinical features, Mohs frozen section results, upstaging, multidisciplinary care, and demographics including age and biological sex. Multidisciplinary care included adjuvant radiation, immunomodulatory or chemotherapeutic medications or augmented care by surgical oncology or head and neck surgery. A combination of Brigham and women’s hospital (BWH) and American Joint Committee on Cancer’s (AJCC) 8th edition staging systems were applied to stage the tumors.

Adult (18 years or older) patients who had undergone MMS for cSCC with debulk specimens between the dates above were included in this retrospective chart review. Medical records must have included: initial biopsy pathology report of cSCC, cSCC debulk specimen pathology report, frozen section pathology report from MMS, cSCC staging information, imaging results (if ordered), and documentation of multidisciplinary care (if appropriate).

At the time of presentation for MMS, our clinical practice uses a broad risk-assessment analysis to capture high-risk cSCC and determine the need for tumor debulk evaluation. This includes immunocompromised patients, size greater than 2 cm, location (ear, lip, and genitalia), aggressive histology (poorly differentiated), recurrence, and perineural involvement. Most tumors with one or more of these characteristics were sent for debulk analysis. A total of 42% of cSCC cases from this period were sent for permanent section analysis. At our institution we primarily use BWH staging criteria to subsequently direct management of cSCCs, although AJCC 8th edition is also employed in parallel.

In this study, we define a clinically meaningful change as any upstage to T2b or higher for BWH criteria, and any upstage to T3 or higher for AJCC 8th edition staging criteria. At our institution, these are changes that would result in an augmented care plan for the patient including consideration for sentinel lymph node biopsy, preoperative or surveillance imaging, or referral to other multidisciplinary services (radiation or medical oncology).

## Results

The average age of patients was 79 years old. There were 12 females and 27 males included in the study. Locations of these tumors included: head and neck (not lip or ear), lip, ear, trunk, and extremities. Two tumors were in high-risk locations. Four patients were immunocompromised. Clinical tumor sizes at MMS presentation ranged from 1.3 cm to 8.0 cm, with 35 tumors being greater than or equal to 2.0 cm. Twelve tumors were poorly differentiated on initial biopsy. Two tumors were recurrent squamous cell carcinomas. One tumor had perineural involvement on initial biopsy (Table I). Postoperative tumor sizes ranged from 1.9 cm to 9.4 cm. The average number of stages to clearance for MMS was 1.7 stages.

**Table I:** Patient and tumor characteristics used to identify specimens sent for debulk analysis

Reason for debulk	High-risk location	Immunocomp-romised	Tumors ≥2.0 cm	Poorly differentiated	Recurrent cSCC	Perineural invasion
Number of tumors	2/39 (5%)	4/39 (10%)	35/39 (90%)	12/39 (31%)	2/39 (5%)	1/39 (3%)

### Brigham and Women’s Hospital

Twenty-six tumors were upstaged by MMS compared with initial biopsy using BWH criteria. Twenty-three tumors were upstaged by debulk specimens compared with initial biopsy using BWH criteria. Of the tumors that were upstaged by MMS, 10 were a clinically meaningful change. Eleven of the tumors upstaged by debulk were a clinically meaningful change. Further care included computed tomography imaging, referral to radiation oncology, thoracic surgery, otolaryngology, and increased surveillance.

Twelve tumors were not upstaged from initial biopsy by either MMS or debulk. Twenty-two tumors were upstaged by both MMS and debulk. MMS upstaged 4 tumors that debulk did not. Debulk upstaged 2 tumors that MMS did not.

Of the 26 tumors that were upstaged by MMS, 17 (51%) were upstaged by size, 0 were upstaged by histology, 3 (12%) were upstaged by invasion beyond subcutaneous fat, 0 were upstaged by perineural invasion, and 6 (23%) were upstaged by a combination of the previous.

Of the 23 tumors that were upstaged by debulk, 12 (52%) were upstaged by size, 1 (4%) was upstaged by aggressive histology, 3 (13%) were upstaged by invasion beyond subcutaneous fat, 0 were upstaged by perineural invasion, and 7 (30%) were upstaged by a combination of the previous.

### AJCC 8th edition

Twenty-five tumors were upstaged by MMS compared with initial biopsy using AJCC 8th edition criteria. Twenty-six tumors were upstaged by debulk compared with initial biopsy using AJCC 8th edition criteria. Twelve of the tumors upstaged by MMS were a clinically meaningful change. Thirteen of the tumors upstaged by debulk were a clinically meaningful change. Further care included the same interventions as above for these patients.

Thirteen tumors were not upstaged from initial biopsy by either MMS or debulk. Twenty-five tumors were upstaged from initial biopsy by both MMS and debulk. MMS upstaged 3 tumors that debulk did not. Debulk upstaged 4 tumors that MMS did not.

Of the 25 tumors that were upstaged by MMS, 17 (68%) were upstaged by size, 0 were upstaged by histology, 4 (16%) were upstaged by deep invasion, 0 were upstaged by perineural invasion, and 4 (16%) were upstaged by a combination of the above.

Of the 26 tumors upstaged by debulk 16 (50%) were upstaged by size, 0 were upstaged by histology, 7 (27%) were upstaged by deep invasion, 0 were upstaged by perineural invasion, 6 (23%) were upstaged by a combination of the above.

Of the 39 tumors included, 21 tumors were upstaged by both BWH and AJCC 8th edition staging criteria during MMS. Twenty tumors were upstaged by both BWH and AJCC 8th edition staging criteria during debulk specimen analysis.

## Discussion

Expert consensus recommendations suggest debulk specimen analysis for select cases but do not have strict criteria, which translates into generalized guideline recommendations.^7^ This includes analysis of high-risk features based on careful selection, on the basis of initial biopsy results and for Mohs excisions, information from tumor debulking specimens (before the first Mohs layer) may be combined with findings on Mohs layers for optimal synoptic reporting and tumor staging.^7^^,^^8^

Nemeh et al^9^ found that permanent sections of cSCC taken during MMS resulted in upstaging of tumors. However, our study with permanent sections examined by a dermatopathologist is contradictory. The results contained in this study are significant in that they offer an alternate view to what has been published earlier on this topic by showing that debulk specimens in this clinical scenario do not add information not able to be gained from a biopsy or MMS layer.

Table II shows that permanent sections from debulk specimens analyzed by a dermatopathologist upstaged 59% and 67% of tumors using BWH and AJCC 8th ed, respectively. This is compared with 67% and 64% upstaged by MMS using BWH and AJCC 8th ed, respectively. A majority of tumors were upstaged by both MMS and debulk histological evaluation. Almost half of these upstaged tumors were clinically meaningful.

**Table II:** Total tumors upstaged from initial biopsy by MMS and debulk analysis, and those resulting in a clinically meaningful change∗

Staging system	BWH	BWH upstaged T2b or higher	AJCC 8th ed.	AJCC 8th ed. upstaged T3 or higher	Tumors captured by both BWH and AJCC 8th ed
Upstaged by MMS	26/39 (67%)	10/26 (38%)	25/39 (64%)	12/25 (48%)	21
Upstaged by Debulk	23/39 (59%)	11/23 (48%)	26/39 (67%)	13/26 (50%)	20

*AJCC*, American Joint Committee on Cancer; *BWH*, Brigham and women’s hospital; *MMS*, Mohs micrographic surgery.

∗ Clinically meaningful change is defined as any upstage to T2b or higher for *BWH* criteria, and any upstage to T3 or higher for *AJCC* 8th edition staging criteria.

The results from this study demonstrate that the information gained from debulk specimens does not provide critical histopathologic information to upstage tumors that cannot be ascertained from the initial biopsy specimen or frozen section of MMS tissue sections. Both MMS and debulk specimens were able to upstage cSCC from the initial biopsy. Table III shows that for both BWH and AJCC 8th edition criteria, most of the upstaging was because of size alone, invasion beyond fat or deep invasion alone, or the combination with other high-risk features. Debulk for permanent section upstaged 1 tumor, with a finding of poor differentiation. No perineural invasion was found on debulk specimens that were not already previously identified on the biopsy specimen or MMS layer. Size and invasion beyond fat or deep invasion are criteria that can be assessed clinically at the completion of MMS and do not require a debulk specimen for assessment. Additionally, the intent with a debulk specimen is not to clear the tumor, therefore analysis of depth is limited on permanent section evaluation. While there are many reasons why size may have been a consistent upstaging feature (inaccurate measurement at the time of biopsy), in our cohort, there was documented tumor growth between initial biopsy and MMS in most cases. In summary, evaluation of a debulk specimen on permanent sections may not result in additional information about the tumor and does not lead to a significant difference in upstaging when compared with frozen section evaluation of MMS margins in our cohort.

**Table III:** Reason for cSCC tumor upstage from initial biopsy by MMS and debulk based on BWH and AJCC 8th edition guidelines

Reason for upstage	Size	Histology	Invasion beyond fat/deep invasion	Perineural invasion	Combination
BWH					
MMS	17/26 (51%)	0	3/26 (12%)	0	6/26 (23%)
Debulk	12/23 (52%)	1/23 (4%)	3/23 (13%)	0	7/23 (30%)
AJCC 8th ed.					
MMS	17/25 (68%)	0	4/25 (16%)	0	4/25 (16%)
Debulk	13/26 (50%)	0	7/26 (27%)	0	6/23 (23%)

*AJCC*, American Joint Committee on Cancer; *BWH*, Brigham and women’s hospital; *cSCC*, cutaneous squamous cell carcinoma; *MMS*, Mohs micrographic surgery.

One reason why debulk specimens may not give additional information about the tumor is the shape and depth of the specimen. In a debulk specimen, all surgical margins are not being examined, but in MMS, they are. Additionally, in a debulk specimen, the depth of tissue taken may not be as great as in MMS layers.

The results of this study are important as they affect the health care cost to the patient, MMS surgeon, and dermatopathologist, and the time for debulk collection and processing. At our institution, Medicare reimbursement for the evaluation of pathology without immunohistochemistry averages $65.15. The time spent on analysis under the microscope is not to be diminished as these are often large specimens that require slow and diligent examination. Our finding that permanent section evaluation of tumor debulk specimens by a dermatopathologist does not provide additional information that cannot be garnered by MMS suggests that the process of obtaining and submitting debulk specimens may not be the most time-effective or best use of health care resources and may not be prudent as a routine practice, although isolated scenarios may still warrant a debulk based on the judgment of the treating surgeon. This is an important area of study, as there are currently no guidelines for which tumors should undergo debulk analysis.

## Conclusion

Our study questions the use of permanent section evaluation of debulk specimens of cSCC obtained during MMS. At our institution, debulk specimens taken during MMS did not result in clinically meaningful information to upstage tumors compared with what can be gained from MMS layers and initial biopsy. Therefore, the practice of taking debulk specimens may not be an appropriate use of time and resources in some institutions and settings. This study highlights the need for more stringent criteria to determine which cSCCs are reasonable to undergo debulk specimen analysis with permanent sections. Further studies are needed to investigate the generalizability of the findings in this study. Limitations to this study include retrospective analysis, a small sample size, and a single institution cohort treated by a single surgeon and practice model.

## Conflicts of interest

None disclosed.
